# Ultrafiltration pretreatment enhances membrane distillation flux, resilience and permeate quality during water recovery from concentrated blackwater (urine/faeces)

**DOI:** 10.1016/j.seppur.2020.117547

**Published:** 2020-12-15

**Authors:** F. Kamranvand, C.J. Davey, L. Williams, A. Parker, Y. Jiang, S. Tyrrel, E.J. McAdam

**Affiliations:** a Cranfield Water Science Institute, Vincent Building, Cranfield University, Bedfordshire MK43 0AL, UK; b Centre for Creative and Competitive Design, Cranfield University, Bedfordshire MK43 0AL, UK; c Centre for Thermal Energy Systems and Materials, Cranfield University, Bedfordshire MK43 0AL, UK

**Keywords:** Urine, Faeces, Sanitation, Single-household, Fouling, Ammonia

## Abstract

In this study, the pretreatment of concentrated blackwater using ultrafiltration (UF) was shown to improve the permeability, selectivity and robustness of membrane distillation (MD) for application to wastewater treatment. Concentrated blackwater comprises urine and faeces, with minimal flushwater added. The faecal contribution increased the soluble organic fraction and introduced coarse and colloidal particles into the urine, which increased resistance to filtration during dead-end UF. Ultrafiltration removed the particulate and colloidal fractions (MW > 500 kDa) from the blackwater, which permitted similar permeability and robustness for MD to that observed with urine (29.9 vs 25.9 kg m^−2^ h^−1^), which comprises a lower colloidal organic concentration. Without UF pretreatment, a higher density organic layer formed on the MD surface (197 vs 70 gCOD m^−2^) which reduced mass transfer, and transformed the contact angle from hydrophobic to hydrophilic (144.9° to 49.8°), leading to pore wetting and a dissipation in product water quality due to breakthrough. In comparison, with UF pretreatment, MD delivered permeate water quality to standards satisfactory for discharge or reuse. This is particularly timely as the ISO standard for non-sewered sanitation has been adopted by several countries at a national level, and to date there are relatively few technologies to achieve the treatment standard. Membrane distillation provides a robust means for concentrated blackwater treatment, and since the energy required for separation is primarily heat, this advanced treatment can be delivered into areas with more fragile power networks.

## Highlights

• UF is used as a pre-treatment to reduce membrane distillation fouling.
• UF removes particles and colloids from blackwater but low MW fraction increases.
• Whilst UF treated blackwater high in organics, flux and water quality is stable.
• Without UF pre-treatment, severe fouling reduces flux, which reduces quickly.
• With UF pre-treatment, permeate quality consistently meets treatment quality.

## 1 Introduction

Membrane distillation (MD) provides a high rejection barrier to organics, including low molecular weight non-volatile solutes [13], inorganics and pathogens, similar to that of reverse osmosis [11], [7]. The hydrophobic membrane used in MD is characterised by a contact angle exceeding 90° and when combined with a small pore diameter, confers a high breakthrough pressure (Δ*P_interface_*) sufficient to repel water [10]:

(1) $$\Delta P_{\mathrm{interface}}=P_{\mathrm{liquid}}-P_{\mathrm{vapour}}=-2B\gamma_{L}\frac{cos\theta }{r_{\max}}$$

where γ_L_ is liquid surface tension, θ is solid-liquid contact angle, B is geometric function describing pore shape, and r_max_ is the maximum pore radius [36]. The repulsion of water creates a vapour liquid interface at the air filled pores, through which the driving force for water vapour mass transport is provided by the vapour pressure difference initiated between the feed and permeate sides of the membrane [21].

To sustain water flux, the gas phase must be retained within the pores. However, liquids with low surface tension (γ_L_) or alterations to the solid-liquid contact angle caused by adsorption or other surface fouling phenomenon (cosθ), can reduce the breakthrough pressure (Eq. (1)), leading to an increased probability for pore wetting. Wetting lowers water productivity and risks a diminution of product water quality [36]. Whilst the character of the foulants can be considered equivalent to those experienced with other membrane technologies applied to the same application, there is a distinction in the fouling mechanisms experienced in MD due to differences in membrane chemistry (e.g. hydrophobicity) and the use of heat rather than pressure as the driving force, which can cause effects such as disaggregation of HMW organics at higher feed temperatures [30]. To illustrate, several authors have proposed that fouling of MD membranes by humic acid (molecular weight 227 g mol^−1^) is less significant than other membrane processes [38], [13]. Higher molecular weight organics such as proteins have been suggested to impose severe fouling, possibly through adsorption induced by multiple charged and amphiphilic moieties resident within the molecular structure [31]. However, the opposite has also been shown [30], the extent of fouling by high molecular weight organic compounds and inorganic contaminants (i.e. scaling) being strongly dependent upon the heat imposed to provide the vapour pressure gradient, perhaps owing to their influence on temperature polarisation at the membrane wall [39].

The potential for membrane distillation to achieve higher Gain Output Ratios (GOR) than conventional distillation methods particularly at smaller process scales, together with the increased process intensification provided by the specific surface area and its viability to facilitate distillation from lower quality heat sources, has increased the commercial prospect for water recovery and zero water discharge applications [9], [35]. Increasing interest has also been shown for its application to wastewater treatment, where waste heat can be used to provide the driving force for separation since this delivers the opportunity to recover higher quality water for a reduced cost to treatment [11]. Whilst the high flow rates for conventional sewage treatment make the energy balance difficult to reconcile, this can be realised in the decentralised treatment of concentrated blackwater [19], where the elimination of flushwater markedly reduces flow to treatment by two orders of magnitude [14]. The present lack of technological options for decentralised blackwater treatment remains a critical barrier in providing proper sanitation to 2.4 billion people globally [40], which leads to approximately 700,000 child deaths per year [5] and economic losses of around $260 billion annually worldwide due to lost productivity and medical cost associated with poor sanitation [16]. Electrochemical oxidation and reverse osmosis (RO) have been considered for concentrated blackwater treatment but require an electrical energy demand of up to 180 Wh capita^−1^ d^−1^ [8] which is not feasible in low-income countries where networked power supplies are extremely fragile, and the cost of power can be prohibitive [17], [4]. Furthermore, separation provided by these individual technologies is insufficient to recover water from blackwater to the same standard as for MD [25], [22], [19] due to the complexity of the wastewater and the broad range of sanitary determinands that must be adhered to. In the case of RO, the osmotic pressure imposed by blackwater is thermodynamically limiting, requiring high ‘head’ pumps to facilitate the driving force for separation which cannot be scaled down and are economically prohibitive. In comparison, distillation technology is scaled on volume and not concentration, making it suitable for the treatment of low volume concentrates to high recovery ratios where the primary thermal energy requirement can be provided by solar thermal [3], biogas [20] or the combustion of the energy rich faecal sludge fraction (24.3 MJ kg^−1^, [32]).

Concentrated blackwater comprises urine and a fraction of the faecal sludge, the latter introducing an organic rich particulate phase which presents a further potential fouling mechanism. Goh et al. [11] reported that whilst a 20 µm biofilm provided some resistance to heat and mass transfer, which slightly modified flux, wetting was not induced, which the authors attributed to the chemical and structural characteristics of the organic matrix. In blackwater, the soluble organic fraction from urine comprises high molecular weight compounds such as human serum albumin (MW 66.4 kDa; [29]) and low molecular weight compounds including urea (CH_4_N_2_O, MW 60.1 g mol^−1^) and bile acids conjugated with an amino acid (e.g. sodium glycocholate (C_26_H_42_NNaO_6_) MW 487.6 g mol^−1^; [23]). These low MW bile salts are responsible for the significant reduction in surface tension (55mN m^−1^ versus 73mN m^−1^ for water) due to their amphiphilic and hydrophobic contributions [28]. Regardless of this reduction in fluid surface tension, Kamranvand et al. [19] did not observe wetting during the membrane distillation of urine even with a membrane of coarse pore radius, indicating reasonable process resilience to this specific organic matrix; these observations support the rationale for adoption of MD for direct potable reuse in space missions [12]. However, faecal contamination of urine did reduce membrane permeability due to the formation of a particle cake which introduced two effects: (i) an initial reduction to heat and mass transfer, which lowered water flux; and (ii) the subsequent introduction of wetting, which diminished water quality. The authors proposed that adoption of a tighter pore size coupled with the introduction of pretreatment, could help restore both mass transport and selectivity properties despite the more challenging feedwater quality. Whilst the use of ultrafiltration as a pretreatment for reverse osmosis is a recognised synergy at industrial scale, the same relationship has been rarely reported for membrane distillation [13] and particularly for such a challenging source water as blackwater. The aim of this study is therefore to establish the significance of ultrafiltration as a pretreatment for membrane distillation to enhance the viability of MD as a solution for decentralised (non-sewered) wastewater treatment. The specific objectives are to: (i) characterise the impact of faecal contamination from blackwater on ultrafiltration productivity and selectivity; (ii) determine the enhancement provided by pretreatment to mass and heat transfer in MD; and (iii) characterise any improvement to the selectivity provided by MD during blackwater treatment through the use of pretreatment.

## 2 Materials and methods

### 2.1 Experimental setup for membrane distillation

**Fig. 1:**
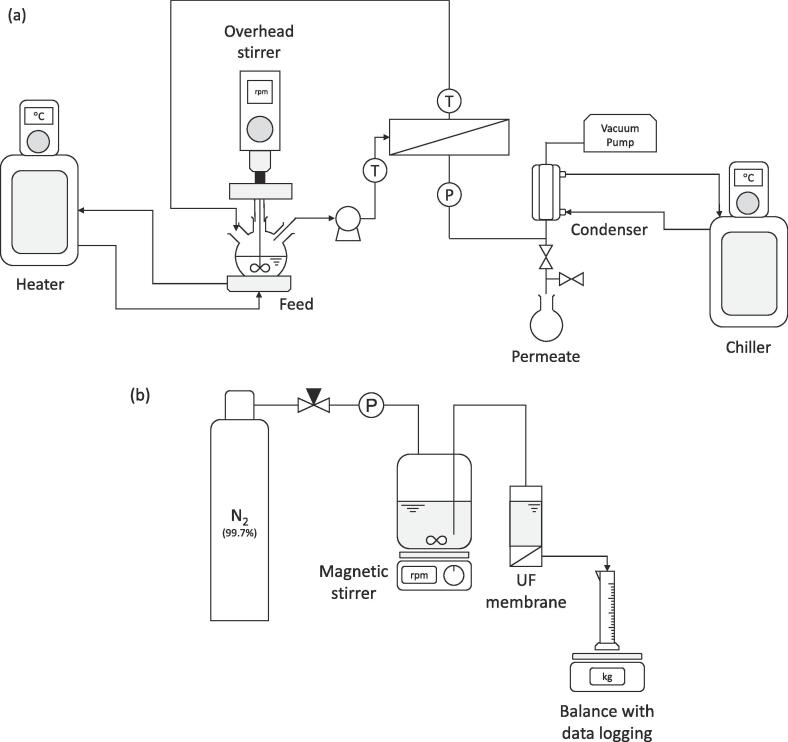
Schematic of the experimental set-ups used in this work (a) vacuum membrane distillation and (b) ultrafiltration.

Feedwater was continuously mixed at 150 rpm using a Tornado™ overhead stirring system (Radley Ltd, Saffron Walden, UK), while heated on a Breeze™ work station (Radley Ltd, Saffron Walden, UK) to 60 °C using a recirculating heater (Huber, Ministat 230 Pilot ONE Controller, Saffron Walden, UK). A Masterflex L/S peristaltic pump (Cole-Parmer, London, UK) recirculated feedwater from the heated flask to the membrane cell at a fixed flow rate (500 mL min^−1^) to sustain a crossflow velocity of 0.1 m.s^−1^ over the membrane surface (Fig. 1a). The plexiglass membrane cell comprises of an unsupported hydrophobic PTFE flat sheet membrane (Cobetter filtration, Hangzhou, China) with nominal pore size of 0.1 µm (4 cm × 14 cm) and thickness of 53 µm, which was placed on a stainless steel mesh support (2.5 cm × 11 cm × 0.02 cm). The open mesh area (and hence active membrane area) was equivalent to 791.4 mm^2^. The stainless steel mesh was sited within a pocket (2 cm × 10 cm, maximum height 5 mm), which was connected to a condenser and a vacuum pump (Vacuubrand, Brackley, UK). Vacuum pressure was measured using a pressure transducers (Omega Ltd., Manchester, UK). The Plexiglass membrane cell comprised a channel (10 cm × 2 cm × 4 mm) which permitted continuous recirculation of the feedwater over the membrane. Feed temperature was measured using k-type thermocouples (LabJack Corporation, Lakewood, USA) and recorded on PC using data acquisition (LabJack Corporation, Lakewood, USA). Permeate was collected using a cold temperature condenser (2 °C) (GPE Scientific, Leighton Buzzard, UK) and a condensation trap (Scientific Glass Laboratories Limited, Stoke-On-Trent, UK). During the experiment, permeate mass was measured temporally on a Symmetry analytical balance (Symmetry PT − 413I PT-Series Precision Toploading Balance, Cole-Parmer, London, UK). Following experimentation, 100 mL deionised water [19] was used to rinse and recover the organic fraction and the reversed organic fraction normalised to membrane surface area:

(1) $$\mathrm{Organic}\;deposition=\frac{C_{\mathrm{COD}}V}{A}$$

where C_COD_ is the concentration in rinse solution (mg L^−1^), V is volume of rinse solution (L) and A is membrane surface area (m^2^). The dimensionless flux (J*) was used to evaluate flux recovery following permeation:

(2) $$J^{*}=\frac{J}{J_{0}}=\frac{J_{\mathrm{clean}}}{J_{\mathrm{final}}}$$

where J, J_0_, J_clean_ and J_final_ are the final flux, virgin flux, clean flux post cleaning and final flux at the end of permeation (kg m^−2^h^−1^).

### 2.2 Experimental setup for dead-end ultrafiltration

Concentrated blackwater was pre-treated using ultrafiltration (Fig. 1b). The system comprised of an 800 mL reservoir (Amicon® Stirred Cell Reservoir, Merk Millipore, Watford, UK) which was connected to a 400 mL Amicon® Stirred Cell (Merk Millipore, Watford, UK) through a selector valve (Merk Millipore, Watford, UK). The reservoir was placed on top of a magnetic stirrer (Magnetic Stirrer H3760, Sigma-Aldrich, Dorset, UK), providing mixing to the feed at 150 rpm. The reservoir was connected to a nitrogen gas supply (BOC, Guildford, UK) to maintain the driving force for pre-treatment. The gas pressure was regulated at 140 mbar using a regulator (type 70BP, Marsh Bellofram Europe Ltd, Nottingham, UK) and gauge (S.M. Gauge Company Ltd, Bristol, UK). Ultrafiltration discs made from PES with 100 kDa NMWCO were used (Merk Millipore, Watford, UK). Filtration proceeded in the absence of mixing (dead-end). Permeate volume was measured in real-time by an analytical Symmetry balance (PT − 413I PT-Series Precision Toploading Balance, Cole-Parmer, London, UK). Total resistance was calculated according to:

(3) $$J=\frac{\Delta P}{\mu R_{T}}=\frac{\Delta P}{\mu \left(R_{m}+R_{f}\right)}$$

where ΔP is the total hydraulic pressure drop across the membrane and fouling layer (Pa), µ is the dynamic viscosity of the permeate (Pa s^−1^) and R_T_, R_m_ and R_f_ are total, membrane and fouling layer resistances respectively (m^−1^).

### 2.3 Preparation of feedwater

Human faeces and urine were collected anonymously in accordance with methods approved through the Cranfield University Ethics review (CURES: 2310/2017; 2407/2017). Subsequently, urine and faeces was mixed at specific gravimetric ratios (w/w) of up to 7 to 1, representative of human production, which was followed by homogenising the mixture at 400 rpm for 20 min, using an overhead stirrer (Hei-TORQUE Precision 400, Heidolph Instruments GmbH, Nuremberg, Germany) to form concentrated blackwater, also described as faecally contaminated urine (FCU). Concentrated blackwater was pre-screened using a 2 mm stainless-steel mesh to remove large particles and prevent tube clogging.

### 2.4 Analytical methods

Ammonium (NH₄^+^-N) and COD were measured by spectrophotometry (Spectroquant® cell tests, Merck Millipore, Watford, UK). Electrical conductivity (EC) and pH were determined using conductivity meter (Mettler Toledo, Leicester, UK) and Testo pH meter (0563 2061, RS Components Ltd., Corby, UK), respectively. The 9215C and 9215D methods together with 9922B and 9922D from the Standard Methods for the Examination of Water and Wastewater (20th Edition, APHA) were used for the E.Coli and Total Coliform analysis. Log reduction of E. Coli was calculated according to:

(4) $$\mathrm{Log}\;reduction\;value,\;R_{\log}=\frac{C_{f}}{C_{p}}$$

where C_f_ and C_p_ are the feed and permeate concentrations respectively. The particle contribution was examined using two methods: (i) Laser diffraction was used to produce a volume based size distribution to characterise coarse particles (Mastersizer 3000, Malvern Analytical Ltd, Malvern, UK); and (ii) the finer particle range (<1.2 µm) was characterised by colloidal fractionation using a continuously stirred cell with a volumetric capacity of 400 mL (Merck Millipore, Watford, UK). Serial fractionation was conducted with agitation above the membrane, whilst under 1 bar Nitrogen pressure, to a 50% product conversion at each fractionation stage to minimise concentration polarisation [26]. Successive membrane discs with NMWCO values of 500, 100, 50, 30, 10, 5, and 1 kDa were used (Polyethersulphone, Merk Millipore, Watford, UK). Clean water flux and membrane contact angle was measured using OCA 25 Contact Angle System (DataPhysics Instruments GmbH, Stuttgart, Germany).

## 3 Results and discussion

### 3.1 Impact of faecal contamination on ultrafiltration permeability and water quality

**Fig. 2:**
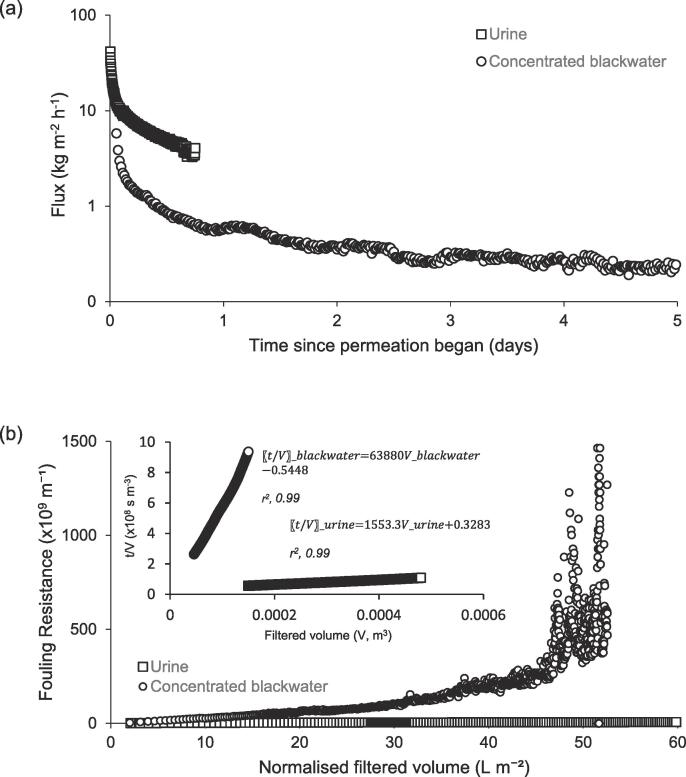
Evaluation of flux during dead-end ultrafiltration of concentrated blackwater: (a) time; and (b) concentration factor. Conditions: Molecular Weight Cut Off, 150 kDa; Pressure, 140 mbar; Feed temperature, 21 °C.

**Fig. 3:**
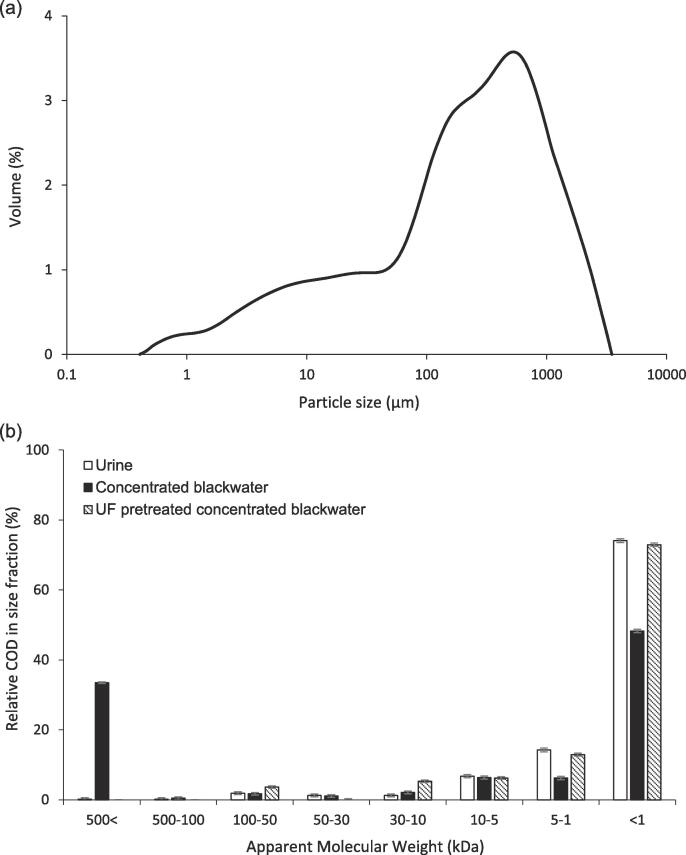
Particle size and colloidal fractionation of Feedwater used during membrane distillation: (a) particle size distribution for concentrated blackwater; (b) colloidal fractionation for all three feedwaters.

Pretreatment of concentrated blackwater (1.8% w/w, faeces/urine) was undertaken using a 100 kDa membrane at 140 mbar, and compared to the filtration behavior of urine as a reference medium (Fig. 2a). The 100 kDa membrane had a clean water permeability of around 753 kg m^−2^h^−1^ bar^−1^. Two stages of filtration were identified, an initial rapid decline in flux, followed by a linear region of filtration. The first stage corresponds to blocking filtration, in which membrane resistance (*R_m_*) controls the rate of flow, whereas the second stage is best described by cake filtration, for which *R_m_* becomes increasingly negligible [6]. The transition from ‘stage 1′ to ‘stage 2′ fouling occurred at around 10 and 1 kg m^−2^h^−1^ for urine and concentrated blackwater respectively. The greater permeability loss in the concentrated blackwater extended the time to filtration (completed at a product water recovery, 50%), terminating in a pseudo steady-state flux approaching around 0.21 kg m^−2^h^−1^. The impact of the inclusion of the particulate fraction on ‘stage 2′ filtration was more evident through analysis of the total resistance to filtration (Fig. 2b). The gradient of the straight-line section of the t/V versus V transformation is analogous to the modified fouling index (Fig. 2b, inset), where the difference in the gradient of the slopes can be directly attributed to the impact of the faecal particles [[37]]. In this study, faecal contamination increased feedwater COD from 3789 to 7340 mg L^−1^ (Table 1, particulate fraction, 3551 mgCOD L^−1^) and was characterised by a broad particle size distribution with a mode of around 500 µm (Fig. 3a); there were insufficient particles in the urine for size determination. The gradient for stage 2 cake filtration of blackwater was around 40 times greater than for urine.

**Table 1:** Separation behaviour determined for dead-end ultrafiltration membrane (150 mbar, 100 kDa PES)) over first five hours of filtration.

		E-Coli	COD	NH4 + -N	pH	EC
		(cfu 100 mL^−1^)	(mg L^−1^)	(mg L^−1^)	(–)	(mS cm^−1^)
*Concentrated blackwater*	cFeed	1.6 × 10^6^	7340	330	7.0	9.7
	Permeate	a<1 × 10^3^	4545 ± 256	539 ± 210	7.9 ± 0.7	11.7 ± 2.6
	dRemoval	b3.2	38%	−38%	N/a	−21%
*Urine*	cFeed	a<1 × 10^3^	3789	205	6.4	N/r
	Permeate	e<10	3736 ± 292	199 ± 17	6.6 ± 0.1	N/r
	Removal	N/a	1.4%	3%	N/a	N/r

N/a – not applicable; N/r – not recorded.

a Equivalent to minimum detection limit (10 cfu ml^−1^) for spread plate method.

b Log reduction value, $R_{\log}=\left(C_{f}/C_{p}\right)$.

c Based on initial concentration.

d As percentage unless otherwise stated.

e Equivalent to minimum detection limit (0.1 cfu ml^−1^, i.e. 10 cfu 100 mL^−1^) for membrane filtration method.

Organics separation was characterised by COD removal of 38% and 1.4% for concentrated blackwater and urine respectively, the difference accounted for by the retention of the particulate fraction during blackwater filtration (Table 1). In addition to the bulk particle fraction, the colloidal and soluble organic fractions were also evaluated (Fig. 3b). Concentrated blackwater broadly comprised a bimodal distribution, with around 30% colloidal organics greater than 500 kDa and around 50% below 1 kDa. Following ultrafiltration, the coarse particles and high MW organics were removed from the concentrated blackwater and the relative MW distribution was more comparable to urine, where the dominant size fraction was < 1 kDa, mostly comprising of bile acids and urea [33]. A 3.2 log reduction value of E-Coli was achieved for blackwater, this value being defined by achieving the method detection limit in the permeate.

### 3.2 Ultrafiltration pretreatment enhances mass and heat transfer in membrane distillation

**Fig. 4:**
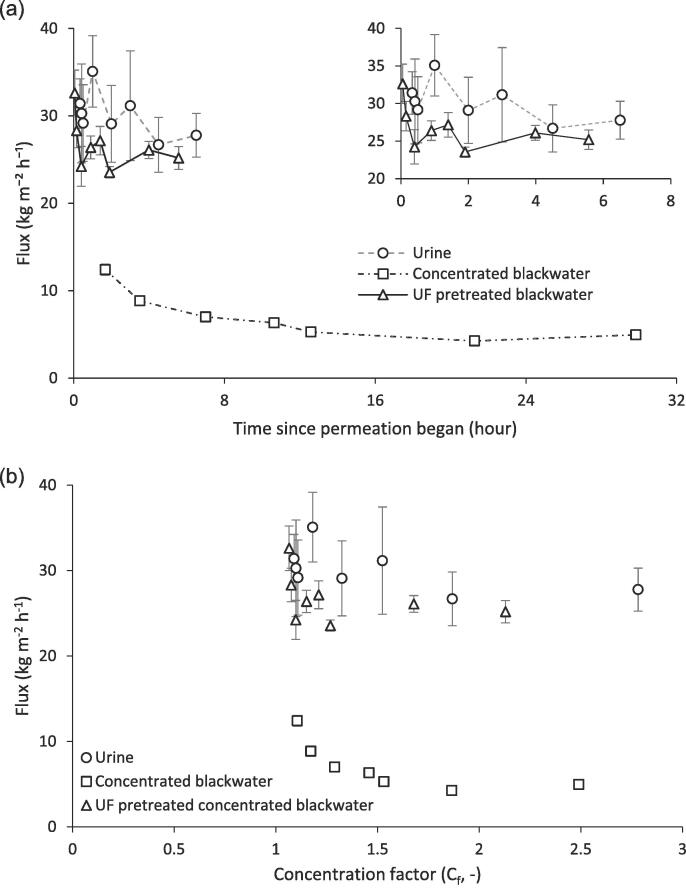
Evaluation of flux during the membrane distillation of urine, concentrated blackwater and UF pretreated concentrated blackwater: (a) time; and (b) concentration factor. Conditions: Vacuum, 48 mbar; Feed temperature, 60 °C; crossflow velocity, 0.1 m s^−1^.

To establish the impact of ultrafiltration as a pretreatment, ultrafiltration treated blackwater was compared to blackwater without pretreatment and urine, using the same boundary conditions and operated to an equivalent water product recovery of 65% (Fig. 4a). An initial flux of 30 kg m^−2^h^−1^ was achieved with urine, which is comparable to the flux achieved with deionised water (Table 2). Membrane flux was reasonably consistent throughout the duration of filtration, despite achieving a concentration factor exceeding 2.5 from a feed solution already comprising a high initial organic concentration (Fig. 4b). Kamranvand et al. [19] identified reasonable flux stability during the membrane distillation of urine but at much lower flux. The higher flux in this study can be accounted for by the thinner membrane wall (53 µm cf. 190 µm) which reduced heat and mass transfer resistance, whereas the improved stability is likely to arise from the smaller pore size adopted (0.1 µm cf. 3 µm stretched pore length). Importantly, in this study, consistent permeation at high concentration factors (or product water recoveries) demonstrates viability, which was endorsed by the use of membrane distillation for water reclamation in limited space applications [12].

**Table 2:** Deposition behaviour and fouling reversibility during membrane distillation with three different source waters.

	Flux			Flux recovery		bRinsed fraction		Contact angle		
	(kg m^−2^h^−1^)			Virgin flux	Used flux	Solution	Deposition	(°)		
	aVirgin	Used	Post-clean	(J/J_0_)	(J_clean_/J_final_)	(mgCOD l^−1^)	(gCOD m^−2^)	Virgin	Used	Post-clean
*Urine*	31	29.9	29.9	0.96	1	65	82	144.9	95.8	119
*Concentrated blackwater*	31	7.7	13.7	0.25	1.8	156	197	144.9	49.8	97
*UF pre-treated concentrated blackwater*	31	25.9	23.6	0.84	0.92	70	88	144.9	62.2	106

a Based on water flux using deionised water; virgin flux for salt water (11.5 kg m^−3^, 0.2 M NaCl, 20 mS cm^−1^) 27 kg m^−2^h^−1^.

b Physical rinse of membrane using 100 mL DI water.

In contrast, a steep decline in membrane flux was observed with concentrated blackwater, leading to a final flux of around 4 kg m^−2^h^−1^, which extended the time to filtration to 30 h in order to achieve the same product water recovery as urine. This implies that the particulate organic fraction conferred an additional resistance. Gryta et al. [13] suggested that, similar to the membrane, the impact of the fouling layer is dependent upon the fouling layer characteristics such as porosity and thickness. For example, a nonporous protein fouling layer may result in thermal and hydraulic resistance while a porous calcium carbonate fouling layer is likely to only contribute to thermal resistance [13], [11]. Organic surface deposition was previously identified by direct visual observation during the membrane distillation of urine [19]. Whilst organic deposition of 82 gCOD m^−2^ was also confirmed in this study for urine, together with a reduction in surface contact angle from 144.9 to 95.8° (Table 2), a decline in flux was not observed which would indicate the structure and thickness of the foulant layer formed presented limited resistance to mass and heat transfer. Physical cleaning post urine treatment also suggested the deposit was not tenacious, since approximately 100% of virgin flux was reestablished. Following blackwater treatment, organic deposition (197 g m^−2^) was more than twice that observed for urine, and reduced the surface contact angle to 49.8°. Whilst physical cleaning improved membrane permeation (J_clean_/J_final_, 1.8), only 44% of initial flux was restored. Goh et al. [11] suggested that within thick organic foulant layers, it is mass transfer rather than heat transfer which limits flux, either through hindered water diffusion due to the high molecular weight organic compounds within the film, or providing physical pore coverage, thus limiting water transport. Through application of ultrafiltration, both the high molecular weight organics and particulate fraction were removed (Fig. 3) and comparable water fluxes to urine were achieved (Fig. 4). The improvement in permeation at high concentration factors by using ultrafiltration pretreatment is also important, given the higher initial organic concentration within the ultrafiltration pretreated concentrated blackwater. This would indicate that it is the character of this low MW organic fraction in the UF permeate rather than the concentration of organics which determines the fouling potential.

### 3.3 Ultrafiltration pretreatment improves membrane distillation permeate quality

**Fig. 5:**
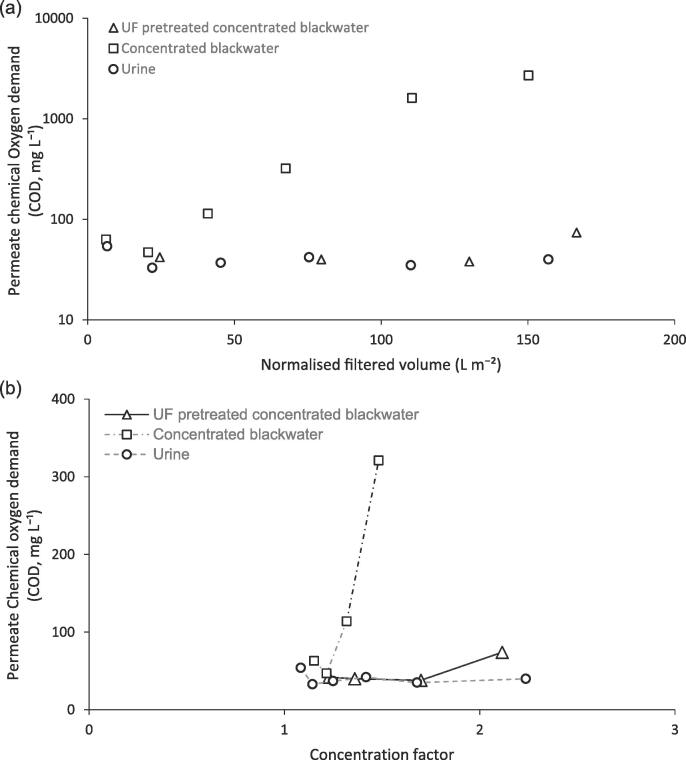
Transient of permeate quality during membrane distillation of three feedwaters: urine, concentrated blackwater and UF pretreated concentrated blackwater: (a) normalised to filter volume; (b) normalised to concentration factor. Vacuum, 48 mbar; PTFE membrane, 0.1 µm; Feed temperature, 60 ⁰C.

For blackwater that has not been pretreated with ultrafiltration, permeate COD rapidly increased after permeating 40 L m^−2^. This can be accounted for by the surface deposition of particulate and colloidal organics which altered the surface contact angle from hydrophobic (>90°) to extremely hydrophilic (49.8°, Table 2). This induced partial wetting of the pores leading to breakthrough of the feed into the permeate [2]. Despite the high COD feed concentrations for urine and ultrafiltration treated blackwater which were 4180 and 7300 mgCOD L^−1^ respectively (Table 3), permeate was consistently below 100 mgCOD L^−1^ (Fig. 5), even with a progressively increasing concentration factor in the feedwater which occurred as a result of constant permeate removal. The recent launch of ISO30500 provides a route to the certification of non-sewered sanitation technologies [1], for which UF-MD can be considered a candidate technology, and contains standards within that must be achieved for discharge or reuse (Table 3). An average COD of 40 ± 7.5 and 49 ± 17 mgCOD L^−1^ for urine and ultrafiltration treated blackwater respectively, indicate the water is nominally sufficient for reuse (threshold, 50 mgCOD L^−1^).

**Table 3:** Separation behaviour determined following membrane distillation (0.1 µm, PTFE; 60 °C feed temperature) of three source waters.

		E-Coli	COD	NH_4_^+^-N	pH	Conductivity
		(cfu 100 mL^−1^)	(mg L^−1^)	(mg L^−1^)	(-)	(mS cm^−1^)
*Urine*	cFeed	a<1 × 10^3^	4180	221	6.33	9.65
	dPermeate	b<10	40 ± 7.5	1 ± 0.5	7.3 ± 0.9	0.3 ± 0.2
	Removal	N/a	98.9%	>99%	–	96.9%
*Concentrated blackwater*	cFeed	6.0x10^7^	7300	398	8.6	11.3
	dPermeate	b<10	75 ± 35	75 ± 94	9.3 ± 0.2	0.6 ± 0.4
	Removal	g6.8	98.9%	81.2%	–	94.7%
*UF pre-treated concentrated blackwater*	cFeed	a<1 × 10^3^	4490	530	8.35	11.5
	dPermeate	b<10	49 ± 17	e104 ± 112	9.57 ± 0.1	0.4 ± 0.3
	Removal	N/a	98.9%	80.4%	–	96.5%
*Proposed standard*		<10	f<50 f<150	70% reduction	6–9	N/a

N/a – not applicable

a Equivalent to minimum detection limit (10 cfu ml^−1^) for spread-plate method.

b Equivalent to minimum detection limit (0.1 cfu ml^−1^, i.e. 10 cfu 100 mL^−1^) for membrane filtration method.

c Based on initial feed concentration (feed concentration increases as permeate is withdrawn).

d Based on first seven hours filtration.

e Below 129 L m^−2^, ammoniacal nitrogen was below 8 mgNH_4_^+^-N l^−1^.

f Tiered threshold, where Category A (<50 mg l^−1^) is for irrigation and other unrestricted urban uses (e.g. toilet flushing), whilst Category B (<150 mg l^−1^) is for direct discharge into the environment.

g Log reduction value, $R_{\log}=\left(C_{f}/C_{p}\right)$.

Ultrafiltration reduced feedwater E. Coli concentration to below the limit of detection (<10 cfu 100 mL^−1^) for urine and ultrafiltration treated blackwater. Whereas the feedwater E. Coli concentration in untreated blackwater was 6.0x10^7^ cfu 100 mL^−1^ (Table 3), membrane distillation recorded a 6.8 log removal, which delivered permeate quality comparable to the reference discharge standard. The high separation factor can be accounted for by several collective mechanisms: (i) size exclusion due to the pore size adopted [7]; (ii) the use of a thermal gradient, and specifically a feed temperature greater than 60 °C, which can inactivate E. Coli [34]; and (iii) the hydrolysis of urea. Following urea hydrolysis, ammonium and hydroxyl ions are liberated which increases pH and shifts the ammonium-ammonia equilibrium toward ammonia above a toxic threshold, consistent with previous literature [18]. Consequently, membrane distillation can be regarded as a robust pathogen barrier for water recovery from blackwater, an increased resilience being provided by the inclusion of ultrafiltration as the pretreatment.

**Fig. 6:**
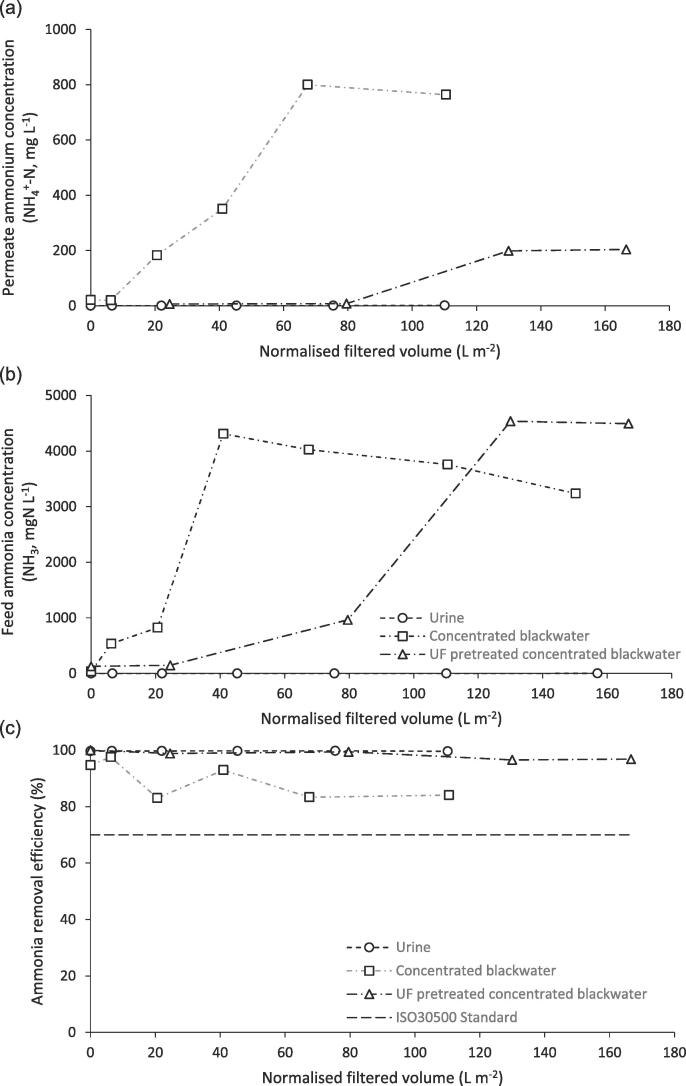
Transient of ammoniacal nitrogen permeate quality during membrane distillation of three feedwaters: urine, concentrated blackwater and UF pre-treated concentrated blackwater: (a) permeate concentration normalised to permeate volume collected; (b) feed ammonia (NH_3_) concentration to evidence transition in nitrogen concentration and shift in ammonia-ammonium equilibrium; and (c) ammonia removal efficiency plotted against ISO standard. Vacuum, 48 mbar; PTFE membrane, 0.1 µm; Feed temperature, 60 ⁰C.

However, the shift in ammonium-ammonia equilibrium increased the relative volatile ammonia fraction which in distillation will reduce the probability for separation (Fig. 6). Such a transformation was not evidenced for urine, as suggested by the low permeate ammonium concentration, which yielded an ammonium removal efficiency exceeding 99%. While urea hydrolysis has previously been shown in urine, the kinetics are slow [41]. Hydrolysis is assumed to proceed extracellularly but will markedly increase with faecal contamination due to the higher pathogen number which can mediate enzymatic activity [24]. This was evidenced by an increase in the feed ‘free’ ammonia concentration (NH_3_, Fig. 6b) for concentrated blackwater and UF pretreated concentrated blackwater. A coincident increase in permeate ammonium concentration resulted (Fig. 6a), where the transient observed is a response to the progressive increase in pH and ammonium concentration in the feed [18]. A lag in permeate ammonium concentration was observed when ultrafiltration was used as the pretreatment for blackwater, where the ammonium permeate concentration was below 8 mgNH_4_^+^-N L^−1^ up to a filtration volume of 129 L m^−2^ (Table 3, Fig. 6b). There are two explanations for the improvement to treatment provided by ultrafiltration: (i) upstream pathogen separation reduces the rate of hydrolysis; and/or (ii) the higher membrane distillation flux reduced fluid residence time such that hydrolysis could not proceed to completion within the processing time. Whilst hydrolysis reduced ammonium removal efficiency for untreated concentrated blackwater, the removal efficiency was above that required by the ISO standard [1]. The modest reduction in feedside ammonia also observed for untreated blackwater, can be ascribed to the volatilisation of ammonia into the feedside gas phase, the loss being reflective of the long processing time imposed by the reduction in membrane flux. More robust design could be facilitated through reducing processing time by increasing membrane surface area, which would constitute a negligible increase in cost based on the small processing volumes per capita within the proposed application (around 1.5 L person^−1^ d^−1^).

## 4 Conclusions

In this study, the pretreatment of feedwaters using ultrafiltration was shown to improve the permeability, selectivity and robustness of membrane distillation for complex wastewaters comprising particulate and colloidal material:

• Evaluation of the resistance to filtration during dead-end ultrafiltration evidenced the significant contribution of the particle fraction that is incorporated through faecal contamination, which introduces both coarse and colloidal particles. Whilst the soluble COD fraction also increases in blackwater above that observed in urine, ultrafiltration effectively removed the particulate and colloidal fractions which enabled analogous permeability and robustness during membrane distillation of blackwater that was observed with urine, and would indicate it is the type of organics rather than the concentration which restricts application of membrane distillation.
• During membrane distillation, the particulate and colloidal fractions within concentrated blackwater were found to develop a higher density organic layer on the membrane that was presumed to reduce heat and mass transfer. Whilst the relative contribution of each of these transport resistances is difficult to ascertain, we suggest mass transfer is primarily constrained by the formation of this dense layer. The result was a reduction in membrane surface area contact angle from hydropohobic to hydrophilic, leading to pore wetting and a dissipation in product water quality due to breakthrough.
• Membrane distillation appears a robust technology for application to urine and when ultrafiltration is provided upstream, is reasonably insensitive to faecal contamination. Extensive faecal contamination was applied within this study to test the robustness of separation; solid–liquid separation could be improved through adopting further upstream interventions such as source separation [41] or post flush source separation [27]. Even with a high level of faecal contamination, permeate water quality was sufficient to achieve the discharge standards contained within the ISO standard for non-sewered sanitation. Further improvements in selectivity can be achieved through limiting faecal contamination within the combined treatment train, and reducing processing time, both of which will limit the opportunity for hydrolysis and so improve upon the robustness of volatiles separation (particularly ammonia), and as a result permeate pH.

Asset creation for large scale sewerage connection with partial treatment of sewage in low income countries would require investment of US$136.5 billion per year, which emphasises that intervention through conventional capital intensive sewered networks is not economically feasible [15]. This study demonstrates that membrane distillation can provide a robust means to deliver decentralised treatment, which can lower the cost to treatment through the avoidance of buried infrastructure, coupled with the use of waste heat for treatment which reduces reliance of treatment on expensive and fragile power networks. This experimental evidence is particularly timely as the ISO standard for non-sewered sanitation has been adopted by more than 18 countries for implementation at a national level, and to date, membrane distillation represents the only process that can achieve compliance to the discharge standards proposed within a single process stage. Whilst the water quality produced by MD is sufficient to meet proposed discharge standards, MD could be complimented with polishing technologies such as GAC to produce water of sufficient quality for reuse, which is particularly important for application within resource constrained environments.

## CRediT authorship contribution statement

**F. Kamranvand:** Conceptualization, Data curation, Formal analysis, Investigation, Methodology, Writing - original draft. **C.J. Davey:** Conceptualization, Data curation, Formal analysis, Methodology, Project administration, Resources, Writing - review & editing. **L. Williams:** Funding acquisition. **A. Parker:** Funding acquisition. **Y. Jiang:** Project administration, Resources. **S. Tyrrel:** Project administration, Writing - review & editing. **E.J. McAdam:** Conceptualization, Data curation, Formal analysis, Funding acquisition, Investigation, Methodology, Project administration, Resources, Supervision, Writing - review & editing.

## Declaration of Competing Interest

The authors declare that they have no known competing financial interests or personal relationships that could have appeared to influence the work reported in this paper.
